# Transfer of the *ph1b* Deletion Chromosome 5B From Chinese Spring Wheat Into a Winter Wheat Line and Induction of Chromosome Rearrangements in Wheat-*Aegilops biuncialis* Hybrids

**DOI:** 10.3389/fpls.2022.875676

**Published:** 2022-06-13

**Authors:** Edina Türkösi, László Ivanizs, András Farkas, Eszter Gaál, Klaudia Kruppa, Péter Kovács, Éva Szakács, Kitti Szőke-Pázsi, Mahmoud Said, Petr Cápal, Simon Griffiths, Jaroslav Doležel, István Molnár

**Affiliations:** ^1^Department of Biological Resources, Centre for Agricultural Research, Eötvös Loránd Research Network, Martonvásár, Hungary; ^2^Institute of Genetics and Biotechnology, Szent István Campus, MATE, Gödöllő, Hungary; ^3^Centre of the Region Haná for Biotechnological and Agricultural Research, Institute for Experimental Botany of the Czech Academy of Sciences, Olomouc, Czechia; ^4^Field Crops Research Institute, Agricultural Research Centre, Giza, Egypt; ^5^John Innes Centre, Norwich, United Kingdom

**Keywords:** bread wheat, *Aegilops biuncialis*, *ph1b* mutant, meiotic chromosome pairing, *in situ* hybridization, chromosome flow sorting, homoeologous recombination

## Abstract

Effective utilization of genetic diversity in wild relatives to improve wheat requires recombination between wheat and alien chromosomes. However, this is suppressed by the *Pairing homoeologous gene, Ph1*, on the long arm of wheat chromosome 5B. A deletion mutant of the *Ph1* locus (*ph1b*) has been used widely to induce homoeologous recombination in wheat × alien hybrids. However, the original *ph1b* mutation, developed in Chinese Spring (CS) background has poor agronomic performance. Hence, alien introgression lines are first backcrossed with adapted wheat genotypes and after this step, alien chromosome segments are introduced into breeding lines. In this work, the *ph1b* mutation was transferred from two CS*ph1b* mutants into winter wheat line Mv9kr1. Homozygous genotypes Mv9kr1 *ph1b*/*ph1b* exhibited improved plant and spike morphology compared to Chinese Spring. Flow cytometric chromosome analysis confirmed reduced DNA content of the mutant 5B chromosome in both wheat genotype relative to the wild type chromosome. The *ph1b* mutation in the Mv9kr1 genotype allowed wheat-alien chromosome pairing in meiosis of Mv9kr1*ph1b*_K × *Aegilops biuncialis* F_1_ hybrids, predominantly with the M^b^-genome chromosomes of *Aegilops* relative to those of the U^b^ genome. High frequency of wheat-*Aegilops* chromosome interactions resulted in rearranged chromosomes identified in the new Mv9kr1*ph1b* × *Ae. Biuncialis* amphiploids, making these lines valuable sources for alien introgressions. The new Mv9kr1*ph1b* mutant genotype is a unique resource to support alien introgression breeding of hexaploid wheat.

## Introduction

Bread wheat (*Triticum aestivum* L.) is an essential component of human nutrition. In terms of global production, it is the third most important crop after rice and maize (FAOSTAT, 2019). The annual wheat production area is 220.89 million hectares, which is ~30% of the total area used to cultivate cereals (FAOSTAT, 2018). Hexaploid wheat (2*n* = 6*x* = 42) comprises three subgenomes A, B, and D (Sears, 1952) which originated from three diploid species. *Triticum urartu* Tumanian ex Gandilyan (2*n* = 2*x* = 14, A^u^A^u^) is considered to be the donor of A genome, *Aegilops speltoides* Tausch (2*n* = 2*x* = 14, SS) is closely related to the putative B genome donor, while *Ae. tauschii* Coss. (2*n* = 2*x* = 14, DD) was the D genome donor (Sears, 1954; Okamoto, 1962). The hexaploid wheat genome resulted from two consecutive interspecific hybridizations and polyploidizations. The first of them occurred between *T. urartu* and a species similar to *Ae. speltoides* 0.3–0.5 million years ago and led to the origin of wild emmer wheat *Triticum turgidum ssp. dicoccoides* (Korn.) Thell. (A^u^A^u^BB, 2*n* = 4*x* = 28; Dvořák et al., 1993; Maestra and Naranjo, 2000). Cultivated emmer wheat *T. turgidum ssp. dicoccon* (Schrank) Thell, evolved from the wild emmer wheat due to human selection. Its hybridization with *Ae. tauschii* ~9,000 years ago gave rise to allohexaploid wheat, *T. aestivum* (Dvořák et al., 1998). However, because only a few genotypes were involved in these allopolyploidization events, genetic diversity of hexaploid wheat is narrow (Feldman and Levy, 2012). Also, domestication and 1,000 of years of cultivation narrowed down genetic variation of wheat (Lubbers et al., 1991; Cox, 1997; Xie et al., 2018; Cheng et al., 2019). One of the biggest challenges for breeders worldwide is to develop efficient allele combinations to produce high-yielding and stress-tolerant cultivars with good quality traits under changing global climate.

A powerful strategy to broaden genetic diversity of wheat is a transfer of new genes and alleles from primary, secondary, and tertiary genepools by interspecific or intergeneric hybridization (Friebe et al., 1996; Molnár-Láng et al., 2015). This approach was used to successfully introduce disease resistance as well as adaptive traits to abiotic stress such as heat, drought, and salinity (Schneider et al., 2008; Kishii, 2019; Darkó et al., 2020). However, the utilization of wild genetic diversity in wheat breeding has been hampered by several factors, including hybridization barriers, hybrid abnormalities, and sterility of F_1_ hybrids (Kishii, 2019). These could be overcome using biotechnological approaches such as hybrid embryo rescue and development of amphiploids after chromosome doubling (Taira et al., 1991; Wulff and Moscou, 2014; Kishii, 2019). Reduced pairing between wheat and alien chromosomes during meiosis brings another level of difficulty, especially in the case of gene transfer from tertiary genepool species (Qi et al., 2007).

Transferred alien chromosome segments can only be utilized in wheat cultivars if they are integrated into the wheat genome as wheat-alien translocations. Among various strategies for producing interspecific chromosome rearrangements (Jiang et al., 1993), the induction of homeologous recombination after the modification of meiotic chromosome pairing is the most preferred (Qi et al., 2007). The main advantage is the genetic compensation of transferred alien chromatin for the missing wheat segment (Jiang et al., 1993). However, chromosome pairing in hexaploid wheat is under strict genetic control, ensuring only the formation of bivalents of homologous chromosomes, while homoeologous chromosomes almost never pair (Okamoto, 1957; Riley and Chapman, 1958). This diploid-like meiotic behavior is a significant barrier against wheat-alien homeologous recombination.

Genetic control of chromosome pairing in wheat consists of suppressing and promoting pairing homoeologous (*Ph*) genes (Sears, 1977). Out of them, the *Ph1* locus located on the long arm of chromosome 5B (Riley et al., 1968) has the strongest suppressing effect on homoeologue chromosome pairing. Another locus (*Ph2*) was mapped to the short arm of chromosome 3D (Mello-Sampayo and Lorente, 1968; Mello-Sampayo, 1971) and another suppressor element with a smaller effect was identified on the homoeologous locus on 3A (Driscoll, 1972; Mello-Sampayo and Canas, 1973). Two additional elements with minor suppressing effects were located on chromosomes 4D and 2D (Driscoll, 1973; Ceoloni et al., 1986). Genes promoting pairing of homoeologous chromosomes were identified on group 2, 3, and 5 chromosomes (Naranjo and Benavente, 2015).

The absence of *Ph1* in 5B nullisomics results in a high frequency of associations between homoeologous chromosomes (Riley and Kempanna, 1963). The use of 5B nullisomic plants is not attractive in introgression breeding programs because of reduced fertility, and an attractive alternative is the use of mutants lacking the *Ph1* locus. A Chinese Spring mutant genotype (*ph1b*) carrying a ~70 Mb deletion at the *Ph1* locus (Dunford et al., 1995) was developed by Sears (1976). Later, other deletion mutants in the *Ph1* locus were developed and utilized (Roberts et al., 1999; Al-Kaff et al., 2008). Apart from the *ph1b* mutation, Sears produced a *ph2a* mutation, which is located on the short arm of chromosome 3D at the position of the *Ph2* locus (Sears, 1982). The pattern of chromosome pairing at meiotic metaphase I in the *ph2b* mutant was similar to that of wild type, and no multivalent formation was detected, while the *ph1b* mutant exhibited extensive multivalent formation (Naranjo and Benavente, 2015). In wheat-alien hybrids, the frequency of homoeologous chromosome associations at metaphase I was low, intermediate, and high in the wild type, *ph2b*, and *ph1b* hybrid genotypes, respectively (Naranjo and Benavente, 2015). Due to the ability of the *ph1b* mutation to induce wheat-alien homoeologous recombination, the Chinese Spring *ph1b* mutant has been applied widely in transferring alien genes from the genera *Aegilops* (Riley, 1968; Liu et al., 2011; Niu et al., 2011; Li et al., 2020), *Secale* (Lukaszewski, 2000), *Hordeum* (Rey et al., 2015), *Haynaldia* (Zhao et al., 2013); *Leymus* (Edet et al., 2018) and *Agropyron* (Copete-Parada et al., 2021).

A serious disadvantage of the *ph1b* mutation in the Chinese Spring background is its poor agronomic performance, such as high plant height, low strength of the stem, low yield, and poor quality traits. Because of this, several backcrosses with advanced wheat lines adapted to the local agro-climatic conditions are necessary before the real agronomic effect of the transferred alien chromosome segment can be evaluated (Li et al., 2020). This process could be avoided by development of new deletions for the *Ph1* region in advanced adapted wheat cultivars. This approach was successfully applied by Al-Kaff et al. (2008) who used γ−irradiation of seeds from hexaploid wheat cultivar Paragon and the produced mutants were used for introgression of wild genetic diversity into wheat (Grewal et al., 2018, 2020; Devi et al., 2019).

The transfer of the *ph1b* deletion on chromosome 5B into an advanced wheat cultivar adapted to local agro-climatic conditions means another choice to eliminate unfavorable traits of Chinese Spring from introgression breeding programs. The winter wheat genotype Martonvásári 9 *kr1* (Mv9kr1) is well adapted to the central European conditions and has better agronomic performance than Chinese Spring (Molnár-Láng et al., 1996). Moreover, it carries the *kr1* and *kr2* crossability genes in recessive homozygous form (*kr1kr1kr2kr2*), making this genotype an ideal crossing partner in alien gene introgression programs (Molnár-Láng et al., 2014). The use of this genotype could facilitate the utilization of wheat-alien recombinants. The present work reports on marker-assisted transfer of *ph1b* deletion chromosome 5B from two Chinese Spring genotypes into the wheat Mv9kr1 line. The resulting M9kr1*ph1b* lines were morphologically characterized and the presence of a chromosome 5B deletion was confirmed by flow cytometric analysis. The lack of the *Ph1* locus and its effect on meiotic chromosome pairing was verified at meiotic metaphase I in F_1_ hybrids of the M9kr1*ph1b* mutant genotype and a tertiary genepool species *Ae. biuncialis* Vis. (U^b^U^b^M^b^M^b^) using genomic *in situ* hybridization (GISH). Finally, the presence of wheat-*Aegilops* chromosome rearrangements was confirmed by GISH in amphiploids obtained by colchicine treatment of the wheat-*Aegilops* F_1_ hybrids.

## Materials and Methods

### Plant Material

Winter wheat (*T. aestivum* L.) line Mv9kr1 containing the recessive crossability gene *kr1* (Molnár-Láng et al., 1996) was used as a female parent with two variants of the Chinese Spring *ph1b* deletion line developed by Sears (1977) as pollinators. One deletion line, designated CS*ph1b*_K, was provided by Professor Bernd Friebe (Kansas State University, Manhattan, KS, United States). The second deletion line, designated CS*ph1b*_N, was provided by Dr. Steve Reader (John Innes Centre, Norwich, United Kingdom).

### Production of Mv9kr1 *ph1b* Lines

**Figure 1:**
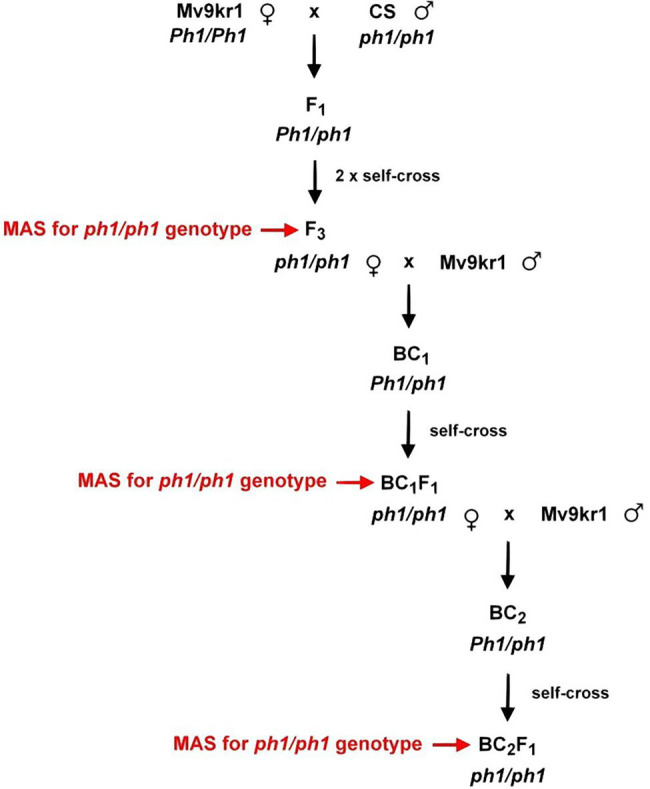
Crossing scheme for development of the Mv9kr1*ph1b* mutant (K and N) lines.

The crossing program for transferring the *ph1b* mutant chromosome 5B from Chinese Spring into Mv9kr1 is summarized in Figure 1. Five spikes (160 florets) were pollinated with CS*ph1b*_K line and another five spikes (148 florets) were pollinated with CS*ph1b*_N line, producing 108 and 128 F_1_ seeds, respectively (Supplementary Table S1).

The Mv9kr1 × CS*ph1b* crosses, as well as the backcrosses with the Mv9kr1, were carried out in the field nursery of MGI ELKH, Martonvásár, Hungary in the 2011–2012 vegetative season. For the self-pollination of marker selected homozygous mutant lines, each of the vernalized (at 4°C for 6 weeks under 20 μmolm^−2^ s^−1^ light intensity) seedlings were planted into 2 L pots filled with a 3:2:1 mixture of garden soil, compost and sand and were grown up in randomized complete block design in glasshouse (Global Glasshouse Venlo). The average day/night temperature was increased from the initial 13/10°C to 23/18°C over 16 weeks, while air humidity was maintained between 60% and 80% by ventilating the glasshouse air. The plants were irrigated weekly to keep the volumetric soil moisture content (VSMC) values between 30% and 35%. The maximum light intensity was gradually increased from the initial 500–700 μmol m^−2^ s^−1^.

The presence of the *ph1b* deletion was confirmed by molecular markers *Xpsr128* and *Xpsr574* specific for the deletion region (Roberts et al., 1999) and used for marker-assisted selection of homozygous *ph1b* plants in F_3_, BC_1_F_1,_ and BC_2_F_1_ generations as described later.

The BC_3_F_1_ seeds of Mv9kr1*ph1b*_K and Mv9kr1*ph1b*_N genotypes have been deposited to the Genebank of the Agricultural Institute, ATK (Martonvásár, Hungary) and are available upon request.

### Evaluation of Morphological Parameters

Morphological parameters (Plant height, Length of the main spike, Spikes per plant, Spikelets per main spike, Seeds per main spike, Seeds per plant) of the wheat line Chinese Spring (CS), parental lines Mv9kr1 and CS*ph1b_*K and CS*ph1b_*N were compared with the BC_2_F_1_ plants of Mv9kr1*ph1b_*K and Mv9kr1*ph1b_*N genotypes. For the morphological evaluation, plants were grown in a glasshouse in the 2020–2021 season. The data representing the mean ± standard deviation of 5–10 plants per genotype for each morphological parameter were compared by Tukey’s *post-hoc* test at *p* < 0.05 where different letters (a–c) indicate significant differences between the genotypes.

### Wheat × *Aegilops biuncialis* Crosses

BC_1_F_2_ Mv9kr1*ph1b*_K genotypes homozygous for the deletion (*ph1b*/*ph1b*) were crossed with *Ae. biuncialis* Vis. (2*n* = 4*x* = 28, U^b^U^b^M^b^M^b^) accessions MvGB380, MvGB382, MvGB1714, MvGB1723, MvGB1733, MvGB1745 MvGB1987 (maintained in the Martonvásár Cereal Genebank) to produce *T. aestivum* × *Ae. biuncialis* F_1_ hybrids (2*n* = 5*x* = 35, ABDU^b^M^b^). As a control for the presence of *Ph1* locus, we also developed Mv9kr1 × *Ae. biuncialis* MvGB1733 F_1_ seeds. The Mv9kr1 × *Ae. biuncialis* MvGB1733 (*Ph1*) and Mv9kr1*ph1b*_K × *Ae. biuncialis* MvGB1733 (*ph1b*) F_1_ hybrids have been used to confirm the positive effect of transferred *ph1b* mutation on wheat-alien homoeologouos chromosome pairing in meiosis. The wheat (Mv9kr1*ph1b*_K) × *Ae. biuncialis* amphiploids (2*n* = 10*x* = 70, AABBDD U^b^U^b^M^b^M^b^) developed by colchicine treatment of the F_1_ hybrids were checked for the presence of wheat-*Ae. biuncialis* chromosome rearrangements by GISH.

### Colchicine Treatment of Mv9kr1 × *Aegilops biuncialis* Hybrids (F_1_ Plants)

The F_1_ seeds were germinated, the seedlings were planted in Jiffy pots with peat pellets of 3 cm in diameter. The young seedlings were vernalized (4°C for 6 weeks under a light intensity of 12 μmol m^−2^ s^−1^ and a day/night period of 10/14 h). Vernalized plants were grown in 2 L pots filled with a 2:1:1 mixture of garden soil, humus, and sand in a phytotron chamber (PGR15, Conviron) until tillering under an initial day/night temperature of 15°C/10°C and 12/12 h light/dark photoperiod. Seedlings at 3–4 leaf stage (Zadoks skale Z24) were removed from the pots and placed into 0.04% (w/v) colchicine for 16 h incubated at 15°C. After the colchicine treatment, the roots were washed under running water for 2 h and the plants were transferred into pots and grown up. Both the day and night temperatures were increased by 2°C after tillering (day length 14 h), stem elongation (16 h illumination), flowering, and 2 weeks after fertilization (Tischner et al., 1997; Türkösi et al., 2018).

### Marker-Assisted Selection of Homozygous *ph1b* Deletion

The *Xpsr128* and *Xpsr574* PCR based markers (Supplementary Table S4), which map within the *ph1b* deletion region (Roberts et al., 1999) were used to confirm the presence of chromosome 5B deletions in Mv9kr1*ph1b_*K and Mv9kr1*ph1b_*N lines. Because the markers are dominant, the absence of their PCR fragments indicates the presence of the *ph1b* deletion in homozygous form, while the presence of their PCR amplicons indicates the presence of *Ph1* locus in heterozygous or homozygous form. The cDNA-based *XAWJL3* PCR marker, which maps to chromosome 2A (Roberts et al., 1999) was used as a positive PCR control (Supplementary Table S4).

Total genomic DNA was extracted from fresh young leaves (plants in the 2-leaf stage) from the wheat line Chinese Spring (CS), parental lines Mv9kr1 and CS*ph1b_*K and CS*ph1b_*N as well as from their F_3_, BC_1_F_1_, BC_2_F_1,_ and BC_3_F_1_ progenies using Quick Gene-Mini80 device (FujiFilm, Japan) together with QuickGene DNA tissue kit (FujiFilm, Japan) according to the manufacturer’s instructions. The PCR reactions were performed in a volume of 15 μl containing 20 ng of template DNA, 1.5 μl of 10× key reaction buffer (MgCl_2_ final concentration of 1.5 mM), 200 μM of each dNTP, 0.2 μM of forward and reverse primers, and 0.375 U of TEMPase Hot Start DNA Polymerase (VWR International, Belgium). The PCR reaction was carried out in Eppendorf Mastercycler (Eppendorf, Hamburg, Germany). The PCR conditions and primer sequences of the three molecular markers were described by Roberts et al. (1999). PCR products were analyzed using a Fragment Analyzer^™^ Automated CE System equipped with a 96-Capillary Array Cartridge (Advanced Analytical Technologies, Ames, United States). The separated PCR products of all genotypes were analyzed and visualized as digital capillary electrophoretic gel images, using the PROsize v2.0 software (Advanced Analytical Technologies, Ames, United States).

### Bivariate Flow Karyotyping

Suspensions of mitotic metaphase chromosomes were prepared from the Kansas and Norwich variants of *ph1b* mutant genotypes (CS*ph1b*_K, CS*ph1b*_N, Mv9kr1*ph1b*_K, Mv9kr1*ph1b*_N) together with wild-type Chinese Spring and Mv9kr1 genotypes according to Vrána et al. (2016). Prior the flow cytometric analysis, GAA microsatellites were labeled by fluorescent *in situ* hybridization in suspension (FISHIS) using 5′-FITC-GAA_7_-FITC-3′ oligonucleotides (Sigma, Saint Louis, United States) according to Giorgi et al. (2013) and the chromosomes were stained by DAPI (4′,6-diamidino 2-phenylindole) at 2 μg/ml. Chromosome analysis and sorting were carried out using FACSAria II SORP flow cytometer and sorter (Becton Dickinson Immunocytometry Systems, San José, United States) as described by Molnár et al. (2016) and Said et al. (2019). Bivariate flow karyotypes FITC-A vs. DAPI-A fluorescence were acquired for each sample. Approximately 3,000 chromosomes were flow-sorted from each 5B chromosome population identified on a flow karyotype onto a microscope slide into a 3.0 μl drop of PRINS buffer supplemented with 2.5% sucrose (Kubaláková et al., 1997). The slides were air-dried and used for fluorescence *in situ* hybridization (FISH). Chromosome identification in the flow-sorted fractions was done after FISH with probes for pSc119.2, Afa family repeat, and 45S rDNA according to Molnár et al. (2016) and Huang et al. (2018). The chromosomes were classified following the karyotype described by Huang et al. (2018).

### Meiotic Chromosome Pairing Analysis

Meiotic chromosome pairing of Mv9kr1 × *Ae. biuncialis* MvGB1733 F_1_ hybrids (2*n* = 5*x* = 35, ABDU^b^M^b^) in the presence (Mv9kr1) and absence (Mv9kr1ph1b_K) of the *Ph1* locus was investigated in metaphase I (MI) of meiosis by means of GISH as described by Molnár and Molnár-Láng (2010). Briefly, anthers containing PMCs at metaphase I were fixed in 1:3 (v/v) acetic acid:ethanol and stored at −20°C for 2 weeks. Then anthers were squashed in 45% acetic acid and slides were stored at 4°C until GISH using M- and U-genomic probes as described below. Images were captured with an AxioImager M2 fluorescence microscope equipped with an AxioCam MRm CCD camera (Zeiss, Oberkochen, Germany) and with appropriate filter sets for DAPI, Alexa Fluor488 and Rhodamine. The images were assembled with AXIOVISION v4.8 software (Zeiss).

In the frame of chromosome pairing analysis at meioic metaphase I, the frequency of meiotic pairing configurations (univalent, bivalent, trivalent, and quadrivalent) and those of scored chromosome associations (w-w, w-M^b^, w-U^b^, M^b^-U^b^) were compared between the wheat × *Ae. biuncialis* MvGB1733 F_1_ hybrids in the presence (Mv9kr1 × *Ae. biuncialis* MvGB1733) and absence (Mv9kr1*ph1b*_K × *Ae. biuncialis* MvGB1733) of the *Ph1* locus. The calculated frequencies represent the percentage of PMCs in which a given pairing configuration or chromosome association was observed. Differences in the mean frequencies of pairing configurations or chromosome associations between the two F_1_ hybrids were investigated by *t*-tests at the *p* = 0.01 significance level.

### Genomic *in situ* Hybridization

Root tips of germinating seeds from the Mv9kr1*ph1b*_K × *Ae. biuncialis* amphiploids containing chromatin introgressed from *Ae. biuncialis* accessions MvGB380, MvGB1714, MvGB1733, MvGB1987, and MvGB1723 were used for chromosome preparation as described by Lukaszewski et al. (2004). Genomic *in situ* hybridization (GISH) experiment was done as described by Molnár et al. (2009). Briefly, total genomic DNAs of *Ae. umbellulata* (UU) and *Ae. comosa* (MM), the diploid progenitors of *Ae. biuncialis*, were labeled with biotin (biotin-16-dUTP; Roche) and digoxigenin (digoxigenin-11-dUTP; Roche) by random priming and used as U- and M-genome probes, respectively. Unlabeled wheat genomic DNA was used as blocking DNA at a ratio of 30:1. Digoxigenin and biotin signals were detected using anti-digoxigenin-rhodamine Fab fragments and Alexa Fluor488, respectively. The slides were evaluated using the Zeiss fluorescence microscope system as described for the meiotic chromosome pairing analysis.

## Results

### Development of the Mv9kr1 *ph1b* Lines

**Figure 2:**
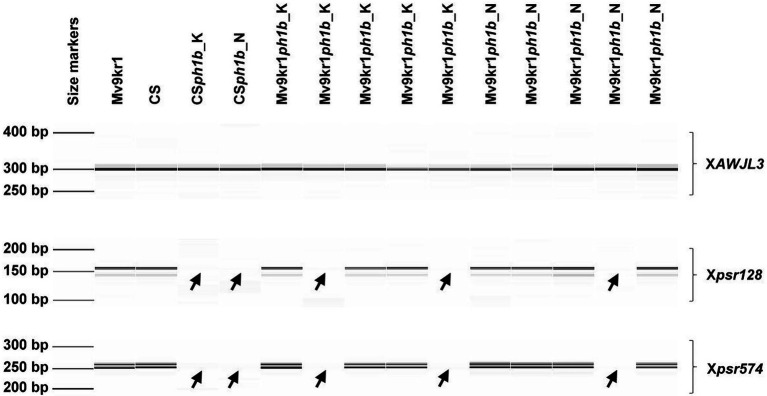
Marker-assisted selection of Mv9kr1*ph1b* mutant genotypes. On the capillary gel electrophoretogram, the presence of PCR amplicons of markers *Xpsr128* and *Xpsr574*, which are specific for the deletion region of chromosome 5B indicate the presence of *Ph1* locus in the wild type wheat genotypes CS and Mv9kr1 and some BC_1_F_1_ Mv9kr1*ph1b*_K and Mv9kr1*ph1b*_N genotypes, while the lack of these amplicons (highlighted by arrows) indicate the presence of *ph1b* deletion in homozygous form in the parental CS*ph1b*_K and CS*ph1b*_N genotypes and in several depicted BC_1_F_1_ genotypes. The marker *XAWJL3*, which mapped to chromosome 2A was used as positive control for the PCR assay.

To transfer the *ph1b* deletion chromosome 5B from Chinese Spring into a winter wheat genotype adapted to the Central European agro-climatic conditions, we crossed the CS*ph1b*_K and CS*ph1b*_N genotypes with the wheat line Mv9kr1 (Figure 1). After two self-pollinations, the F_3_ plants were screened for the presence of the *ph1b* deletion in homozygous state by PCR markers *Xpsr128* and *Xpsr574* specific for the deleted region (Roberts et al., 1999). Homozygous *ph1b* plants were then selected for the morphological characteristics of the Mv9kr1 genotype (small plant height, long spikes), backcrossed with Mv9kr1 (BC_1_ generation), and then self-pollinated to fix the deletion in homozygous state (BC_1_F_1_ generation). BC_1_F_1_ plants were also filtered using PCR for the homozygous *ph1b* deletion (Figure 2) and selected for the morphological traits of the Mv9kr1 parent (plant height, spike architecture). The backcrossing and selection cycle was repeated to produce BC_2_F_1_ plants. The information on the number of seeds analyzed in F_3_, BC_1_F_1,_ and BC_2_F_1_ generations by molecular markers and those of carrying the *ph1b* deletion in homozygous form is summarized in Supplementary Table S2. The frequency of *ph1b*/*ph1b* individuals varied from 12% to 37%. No correlation was found between the frequency of the homozygous deletion and the generation analyzed, nor between the frequency of the deletion and their origin (CS*ph1b*_K or CS*ph1b*_N).

Morphology of the newly developed BC_2_F_1_ Mv9kr1*ph1b* mutants (Mv9kr1*ph1b*_K, Mv9kr1*ph1b*_N) was compared with the wild type genotypes (Mv9kr1, Chinese Spring) and the parental Chinese Spring genotypes carrying the *ph1b* deletion (CS*ph1b_*K, CS*ph1b*_N; Table 1). Wild-type and mutant Mv9kr1 plants were shorter than the CS wheat lines (CS, CS*ph1b* K, and CS*ph1b* N). Apart from plant height, the mutant and wild type Mv9kr1 plants had longer spikes with more spikelets than Chinese Spring (Table 1), indicating that the morphological parameters of the plants carrying the *ph1b* deletion were improved after the transfer into the Mv9kr1 line.

**Table 1:** Morphological parameters of the wheat cv. Chinese Spring (CS), wheat lines Mv9kr1 and CS*ph1b_*K and N mutants and the BC_2_F_1_ Mv9kr1*ph1b_*K and Mv9kr1*ph1b_*N genotypes.

Genotype	Plant height (cm)	Length of the main spike (cm)	Spikes/plant	Spikelets/main spike	Seeds/main spike	Seeds/plant
CS	70.3 ± 4.7^a^	6.7 ± 0.6^c^	3.6 ± 1.3^a^	17.3 ± 1.3^b^	32.8 ± 4.2^a^	107 ± 31^a^
CS*ph1b*_K	71.0 ± 5.0^a^	6.3 ± 0.4^c^	4.5 ± 0.8^a^	17.6 ± 1.8^b^	21.3 ± 5.6^b^	64 ± 11^b^
CS*ph1b*_N	68.5 ± 4.1^a^	6.8 ± 0.4^c^	3.9 ± 1.0^a^	16.9 ± 1.7^b^	20.5 ± 8.1^b^	60 ± 23^b^
Mv9kr1	58.2 ± 5.3^b^	9.6 ± 1.0^a^	3.4 ± 0.9^a^	23.2 ± 0.8^a^	42.2 ± 3.9^a^	134 ± 25^a^
Mv9kr1*ph1b*_K	61.4 ± 2.7^b^	8.1 ± 0.9^b^	3.5 ± 0.8^a^	20.8 ± 1.4^a^	32.4 ± 8.1^a^	103 ± 16^a^
Mv9kr1*ph1b*_N	61.4 ± 3.8^b^	8.4 ± 1.5^ab^	3.6 ± 1.1^a^	20.8 ± 1.6^a^	14.0 ± 4.5^b^	62 ± 12^b^

Data represent mean ± standard deviation of 5–10 plants per genotype for each morphological parameter. Different letters indicate significant differences between the genotypes at *p* < 0.05, using Tukey’s *post-hoc* test. The plants were grown in a glasshouse during the 2020–2021 season.

**Figure 3:**
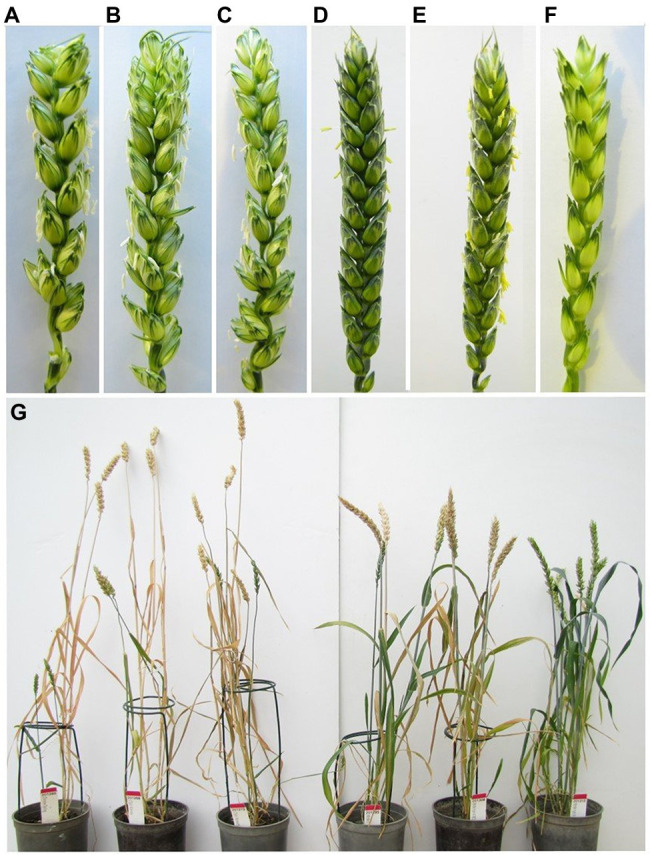
Spike morphology of the wild-type (*Ph1*) plants and of those carrying the *ph1b* deletion: **(A)** wild-type Chinese Spring; **(B)** Chinese Spring *ph1b*_K; **(C)** Chinese Spring *ph1b*_N; **(D)** wild-type Mv9kr1; **(E)** Mv9kr1 *ph1b*_K; **(F)** Mv9kr1 *ph1b*_N; **(G)** Whole plant morphology of the same genotypes (in the same order as it was shown for spike morphology).

Interestingly, the genotype Mv9kr1*ph1b*_K exhibited significantly higher fertility as judged by the higher number of seeds per main spike and seeds per plant as compared to the two CS*ph1b* mutant genotypes. In contrast, the Mv9kr1*ph1b*_N plants differed significantly from the wild type and the other *ph1b* mutant Mv9kr1 lines as they had lower seed set similar to the CS*ph1b* wheat lines. The seed number data indicate that parallel with the plant and spike morphology, the fertility was also improved when the *ph1b* mutant 5B chromosome was transferred from the Kansas CS*ph1b* genotype into Mv9kr1, while the fertility remained low when the mutant chromosome 5B was transferred from the CS*ph1b* Norwich variant. Plant and spike morphology of the CS and Mv9kr1 genotypes carrying the *Ph1* locus or *ph1b* deletion are shown in Figure 3.

### Comparison of Chromosome 5B Size in the Wild Type and *ph1b* Mutant Lines

**Figure 4:**
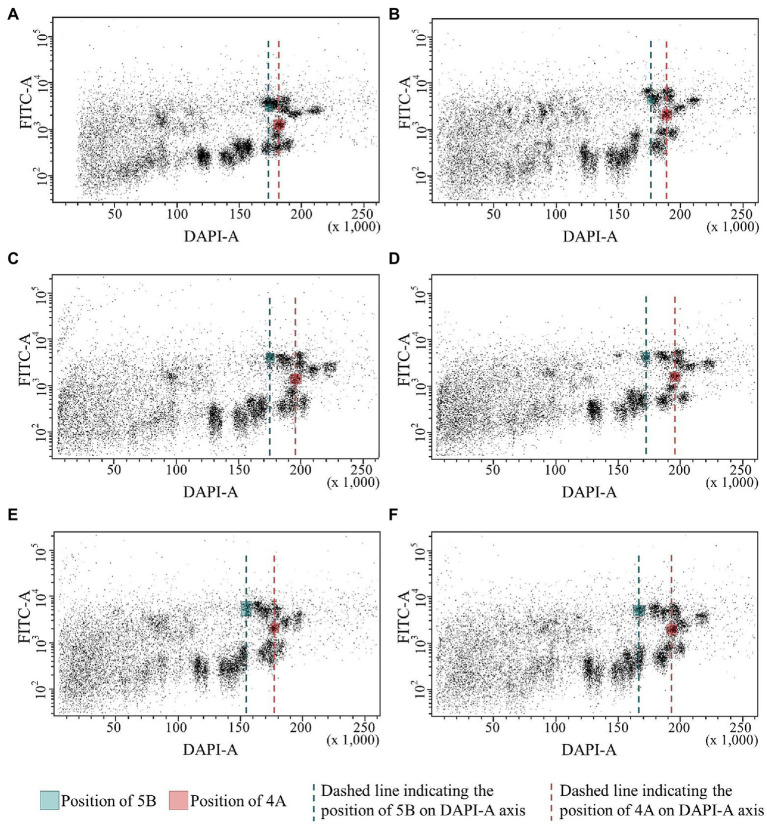
Bivariate flow cytometric analysis (flow karyotyping) of chromosomes isolated from wild-type and *ph1b* mutant wheat lines allows discrimination between the populations of wild-type and deleted chromosome 5B (green line) and chromosome 4A, which served as a reference (red line). Note that the populations of wild-type chromosome 5B in Chinese Spring **(A)** and Mv9kr1 **(B)** lines are closer to the position of 4A population on the DAPI axis. Chromosome 5B populations in CS*ph1b*_K **(C)** and CS*ph1b*_N **(D)** lines have lower DAPI fluorescence intensity and hence are more separated from chromosome 4A population. The same difference between the positions of 5B and 4A chromosomes was detected in Mv9kr1*ph1b*_K **(E)** and Mv9kr1*ph1b*_N **(F)** genotypes.

We used bivariate flow cytometric analysis of suspensions of isolated mitotic chromosomes to confirm the 70 Mb *ph1b* deletion on chromosome 5B in the Chinese Spring and Mv9kr1 lines. Simultaneous analysis of GAA-FITC and DAPI fluorescence permits discrimination of almost all 21 chromosomes of bread wheat, including chromosome 5B, and is sensitive enough to detect changes in chromosome DNA content (Doležel et al., 2021). To highlight changes in the position of chromosome 5B on a dot-plot (flow karyotype) GAA-FITC vs. DAPI, we used the position of chromosome 4A as a reference (Figure 4). Bivariate flow karyotyping of the wild type (*Ph1/Ph1*) Chinese Spring and Mv9kr1 wheat showed that the populations representing chromosome 5B were located close to other B-genome chromosomes (1B, 4B, 7B) which possess large clusters of GAA microsatellite (Figures 4A,B). The difference in DNA content between chromosomes 5B and 4A was small as reflected by small difference in relative DAPI fluorescence (Figures 4A,B). The identity of chromosome 5B population was confirmed by FISH on a chromosome fraction flow-sorted onto a microscope slide. Chromosome 5B was the most frequent in the sorted fraction (52.1% and 57.9% in CS and Mv9kr1, respectively), followed by 1B (39.3% and 39.4%), 4B and 7B (1%–5%; Supplementary Figure S1; Supplementary Table S3). The position of chromosome 5B population shifted to lower DAPI fluorescence intensity, resulting in a greater distance between chromosomes 5B and 4A on bivariate flow karyotypes of Chinese Spring *ph1b* mutant genotypes (CS*ph1b*_K, CS*ph1b*_N) relative to the wild-type plants. These changes reflected lower DNA content of the *ph1b* mutant 5B chromosome in these genotypes (Figures 4C,D).

Interestingly, a bigger shift in the position of the 5B population on a flow karyotype was observed for the Norwich variant of CS*ph1b* mutant as compared to CS*ph1b* from Kansas. Due to this, the *ph1b* chromosome 5B could be discriminated better from the remaining B-genome chromosomes. This was reflected by higher purity in sorted chromosome fractions and the chromosome could be sorted in higher purity (97.2%) from the CS*ph1b*_N genotypes than those from the Kansas variants (CS*ph1b*_K: 89.1%; Supplementary Figure S1; Supplementary Table S3).

Difference in the DNA content between the Kansas and Norwich variants of *ph1b* deletion 5B chromosome were confirmed by flow karyotyping the Mv9kr1 mutants (Figures 4E,F). Similar to the CS mutants, a bigger shift in the position of chromosome 5B population on flow karyotype was observed for the Mv9kr1*ph1b_*N genotype. Consequently, the chromosome was sorted at higher purity (98.5%) as compared to Mv9kr1*ph1b*_K (85.8%).

### Functional Verification of the Mv9kr1 *ph1b* Mutant

In order to verify that the promoting effect of *ph1b* deletion transferred to the Mv9kr1 genetic background on homoeologous chromosome pairing and recombination, we produced wheat × *Ae. biuncialis* F_1_ hybrids. Some of the F_1_ hybrids were used for chromosome pairing analysis at meiotic metaphase I, while other F_1_ hybrid plants were treated by colchicine to produce amphiploids. Because of higher fertility, only Mv9kr1*ph1b*_K genotype was used for the crosses with five accessions of *Ae. biuncialis*. The results of the Mv9kr1*ph1b_*K mutant × *Ae. biuncialis* crosses are summarized in Table 2.

**Table 2:** Number of F_1_ progenies obtained from Mv9kr1*ph1b*_K × *Aegilops biuncialis* crosses and the amphiploid seeds obtained by colchicine treatment of the F_1_ hybrids.

Crossing combination	No. of F_1_ progenies	No. of F_1_ plants treated with colchicine	No. of obtained amphiploid seeds
Mv9kr1 × *Ae. biuncialis* MvGB 1733	20	–	–
Mv9kr1*ph1b_K* × *Ae. biuncialis* MvGB 1733	101	40	12
Mv9kr1*ph1b_K* × *Ae. biuncialis* MvGB 1987	314	50	26
Mv9kr1*ph1b_K* × *Ae. biuncialis* MvGB 1714	247	40	6
Mv9kr1*ph1b_K* × *Ae. biuncialis* MvGB 1723	33	10	4
Mv9kr1*ph1b_K* × *Ae. biuncialis* MvGB 380	89	40	1

**Figure 5:**
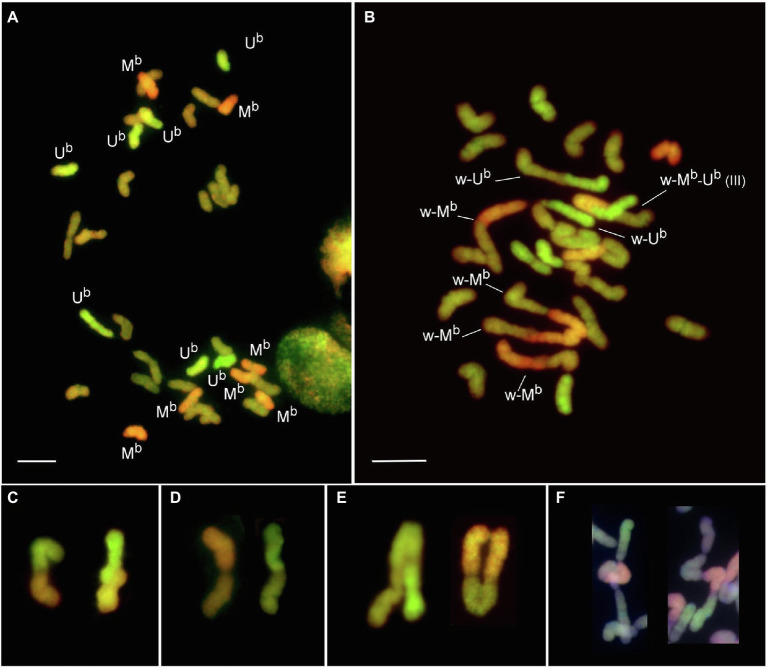
Genomic *in situ* hybridization of PMCs at MI of bread wheat × *Aegilops biuncialis* hybrids (2*n* = 5*x* = 35, ABDU^b^M^b^) in the presence (Mv9kr1) or absence (Mv9kr1*ph1b*_K) of the *Ph1* locus. **(A)** MI cell of an Mv9kr1 × *Ae. biuncialis* MvGB1733 hybrid with functional *Ph1* showing the whole chromosome complement, as seven M^b^ (red), seven U^b^ (green), and 21 unlabeled wheat (brown) univalents. **(B)** MI cell of an Mv9kr1*ph1b*_K × *Ae. biuncialis* MvGB1733 hybrid with seven rod bivalents; four of them involves wheat and M^b^ chromosomes (w-M^b^), three involves wheat and U^b^ chromosomes (w-U^b^) and one involves wheat chromosomes. A trivalent involving wheat, M^b^ and U^b^ chromosomes was also labeled (III). **(C–F)** Selected meiotic pairing configurations: U^b^-M^b^ rod bivalents **(C)**, wheat-U^b^ and wheat-M^b^ rod bivalents **(D)**, trivalents involving wheat and U^b^ or M^b^ chromosomes **(E)**, multivalents involving U^b^, M^b^, and wheat chromosomes **(F)** (in this figure, chromosomes were counterstained with DAPI “blue”). Scale bar 10 μm.

We used GISH to investigate meiotic pairing behavior of the Mv9kr1 × *Ae. biuncialis* MvGB1733 F_1_ hybrids in the presence (Mv9kr1) or absence (Mv9kr1*ph1b*_K) of the *Ph1* locus (Figure 5). The analysis of the pollen mother cells (PMCs) confirmed that the examined hybrids had 21 wheat and 7 U^b^ and 7 M^b^ *Aegilops* chromosomes, corresponding to genome composition of hexaploid wheat × *Ae. biuncialis* F_1_ hybrids (2*n* = 5*x* = 35, ABDU^b^M^b^). As expected, the level of MI chromosome paring was higher in Mv9kr1*ph1b*_K × *Ae. biuncialis* hybrids than in those obtained with wild-type Mv9kr1 genotype (Table 3). We observed significantly higher frequency of rod bivalents, trivalents, and multivalents in the presence of *ph1b* mutation and the increased frequency of chromosome pairing was manifested at the level of chromosome associations (Table 4). Four categories of chromosome associations were scored: associations between wheat chromosomes (w), interspecific associations between wheat and *Aegilops* chromosomes (M^b^ or U^b^), and between *Aegilops* chromosomes. The results of the *t*-test (Table 5) showed that wheat chromosomes paired most frequently with each other, but there was no statistical difference between the wheat-wheat (w-w) chromosome associations and the associations between wheat and the M^b^ genome chromosomes of *Aegilops* (w-M^b^; Tables 4, 5). The number of w-U^b^ and particularly M^b^-U^b^ associations was significantly lower than w-w and w-M^b^ associations. The pairing frequency of *Aegilops* M^b^ and U^b^ genome chromosomes with those of wheat could thus be ranked as follows: w-w = w-M^b^ > w-U^b^ = M^b^-U^b^.

**Table 3:** Frequency of meiotic configurations at metaphase I in bread wheat (Mv9kr1) × *Aegilops biuncialis* MvGB1733 hybrids in the presence (Mv9kr1) and absence (Mv9kr1*ph1b*_K) of *Ph1* locus.

					MI pairing configuration^a^			
Hybrid	PMCs
		I		II		III		IV	
		Total	Mean/cell	Total	Mean/cell	Total	Mean/cell	Total	Mean/cell
Mv9kr1 × *Ae. biuncialis*	56	1,902	33.96	29	0.51	0	0	0	0
Mv9kr1*ph1b*_K × *Ae. biuncialis*	39	609	15.22^**^	253	6.32^**^	75	1.875^**^	6	0.15^**^

a I, univalent; II, bivalent; III, trivalent; IV, quadrivalent.

** Significant difference between the two F1 hybrids at the *p* = 0.01 significance levels.

**Table 4:** Frequency of MI homoeologous associations in bread wheat (Mv9kr1) × *Aegilops biuncialis* MvGB1733 hybrids.

				MI associations				
Hybrid	PMCs
		w-w		w-M^b^		w-U^b^		M-U^b^	
		Total	Mean/cell	Total	Mean/cell	Total	Mean/cell	Total	Mean/cell
Mv9kr1 × *Ae. biuncialis*	56	27	0.48	2	0.03	0	0	0	0
Mv9kr1*ph1b*_K × *Ae. biuncialis*	39	174	4.35^**^	142	3.55^**^	63	1.575^**^	41	1.025^**^

** Significant difference between the two F_1_ hybrids at the *p* = 0.01 significance levels.

**Table 5:** Results of *t*-tests describing differences in the means of various associations involving wheat (w) and *Aegilops* (M^b^, U^b^) chromosomes in the Mv9kr1*ph1b*_K × *Ae. biuncialis* MvGB1733 F_1_ hybrid.

	*t*-value	Value of *p*
w-w/w-M^b^	1.785	0.07836
w-w/w-U^b^	6.659	8.3238E^−9^^**^
w-w/M^b^-U^b^	8.371	2.4886E^−11^^**^
w-M^b^/w-U^b^	5.919	9.3851E^−8^^**^

** Significant difference between the two chromosome associations at *p* = 0.01 significance level.

**Figure 6:**
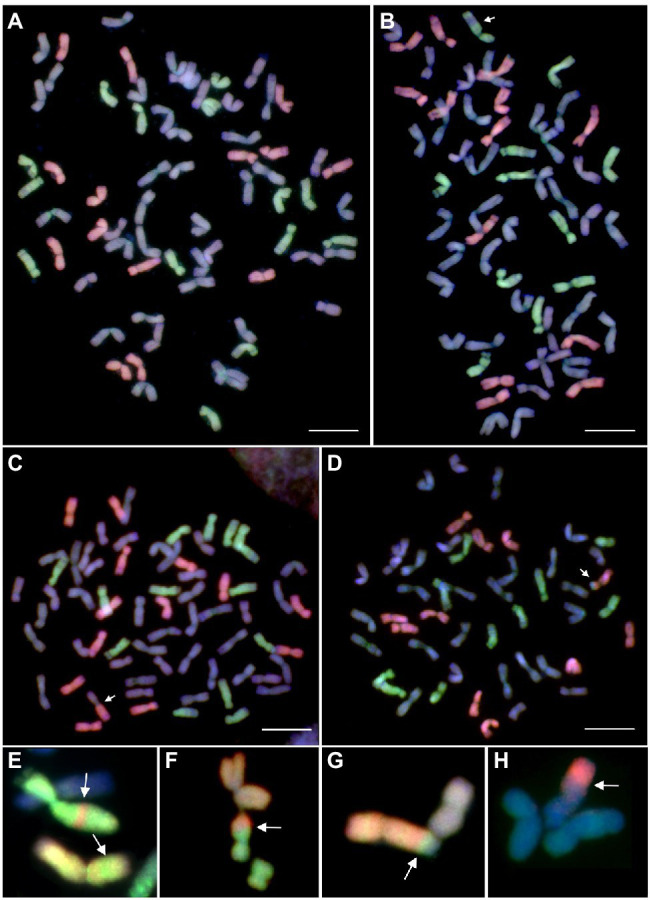
Mitotic metaphase plates of Mv9kr1*ph1b_*K × *Aegilops biuncialis* amphiploid after GISH with differentially labeled M- and U-genomic probes allowing the discrimination of *Ae. biuncialis* M^b^—(red) and U^b^—genome (green) chromosomes from those of unlabeled wheat chromosomes (blue). Partial amphiploid cell without intergeneric recombinant chromosomes **(A)**, a partial cell of 201,226 amphiploid carrying an U^b^-wheat intercalary translocation **(B)**, a cell of genotype 201,246 carrying a wheat-M^b^ Robertsonian translocation **(C)**, and a cell of genotype 201,216 carrying an M^b^-wheat terminal translocation **(D)**. Reciprocal intercalary **(E)** and terminal translocations **(F-H)** detected in additional amphiploids (201,225, 21,413, 201,245, and 21,407, respectively). The recombinant chromosomes are indicated by arrows. The chromosomes were counterstained with DAPI (blue). Scale bar = 10 μm.

We also investigated mitotic chromosome spreads in 24 Mv9kr1*ph1b_*K × *Ae. biuncialis* amphiploids containing *Aegilops* genetic variation from five accessions (Table 2) by GISH in order to check if the increased level of wheat-*Aegilops* meiotic chromosome pairing resulted in interspecific translocations (Figure 6). The GISH analysis of the mitotic cells showed that chromosome number in most of the examined amphiploids were close to the maximum of 42 wheat and 14 U^b^ and 14 M^b^ *Aegilops* chromosomes, which corresponded to the genome composition of the hexaploid wheat × *Ae. biuncialis* amphiploids (2n = 10× = 70, AABBDDU^b^U^b^M^b^M^b^). Seven (29.16%) out of the 24 amphiploid genotypes investigated contained different types of translocations (Robertsonian, terminal and intercalary) between wheat and *Aegilops* chromosomes (Table 6).

**Table 6:** Genomic constitution of Mv9kr1*ph1b_*K × *Aegilops biuncialis* amphiploids.

		No. of Chrs. (mean)				Detected translocation	
Amphiploid combination^*^	No. of plants				Tr./plants		
		w	M^b^	U^b^		Plant ID	Type
Mv9kr1*ph1b*_K × MvGB380	2	42	14	13	0.5	21,407	wheat-M^b^ terminal
Mv9kr1*ph1b*_K × MvGB1723	3	42	14	14	0	–	–
Mv9kr1*ph1b*_K × MvGB1714	3	41.6	14	13.6	0.66	201,245	M^b^-wheat disomic terminal
						201,246	wheat-U^b^ Robertsonian
Mv9kr1*ph1b*_K × MvGB1733	5	41	11.5	12.7	0.2	201,216	M^b^-U^b^ terminal
Mv9kr1*ph1b*_K × MvGB1987	11	40.4	13	13.5	0.27	201,225	U^b^-M^b^ and M^b^-U^b^ reciprocal intercalary
						201,226	U^b^-M^b^ intercalary
						21,413	U^b^-M^b^ terminal

The genomes of wheat (w) and those of *Aegilops biuncialis* (M^b^, U^b^) were discriminated by GISH in the amphiploids produced by different accessions of *Ae. biuncialis*. The mean chromosome number as well as the frequency of intergenomic translocations (expressed by translocations per plant; Tr./plants) were determined for each amphiploid combination. Type of intergenomic translocations detected in different plants is also summarized.

* Amphiploids originated from cross of wheat Mv9kr1*ph1b*_K and *Ae. biuncialis* accessions maintained in the Martonvásár Cereal Genebank (MvGB).

## Discussion

The Chinese Spring *ph1b* mutant produced by Sears (1977) has been used widely in homoeologous recombination-based chromosome engineering in wheat. However, due to poor agronomic performance of Chinese Spring, especially under Central European climatic conditions, the utilization of wheat-alien translocations requires several backcrosses with elite wheat genotypes adapted well to the local agro-climatic conditions. To overcome difficulties related to poor agronomic traits of Chinese Spring, wild type and newly developed *ph1*-mutant variants of hexaploid spring wheat cultivar “Paragon” (Al-Kaff et al., 2008), an elite line in United Kingdom environment, was chosen as key parent for a pre-breeding program in United Kingdom^1^ (Moore, 2015) to introgress chromatin of *Thinopyrum bessarabicum*, *Triticum timopheevii*, and *Aegilops caudata* into wheat (Grewal et al., 2018, 2020; Devi et al., 2019). Using an Axiom 35 K SNP array, the authors also demonstrated the effectivity of high resolution genotyping to detect alien introgressions in wheat (King et al., 2017).

Another approach is the transfer of original *ph1b* deletion from Chinese Spring into a wheat cultivar with better agronomic characters. Using this approach Li et al. (2020) transferred the *ph1b* deletion into a hexaploid spring wheat cultivar Shumai 126, indicating that morphological characters of the *ph1b* mutant lines could be improved by changing the wheat genetic background. Our work extended this this approach to a winter wheat genotype to develop a *ph1b* mutant genotype adapted to the Central European climate. We applied marker-assisted and phenotypic selection for morphological characters (low plant height, long spikes, and improved grain yield) to introduce the *ph1b* deletion into the winter wheat genotype Mv9kr1. Because of the good winter hardiness of Mv9kr1 (Molnár-Láng et al., 1996), the crossing programs with the Mv9kr1*ph1b* mutant plants can be performed under cost-effective field conditions. The wild type Mv9kr1 genotype has been used as crossing partner to introgress chromosome segments from barley (Szakács and Molnár-Láng, 2007, 2010), rye (Szakács et al., 2020), *Thinopyrum* (Kruppa and Molnár-Láng, 2016) and *Aegilops* (Schneider et al., 2005; Molnár et al., 2009; Farkas et al., 2014; Kruppa and Molnár-Láng, 2016) into wheat. The new wheat genotype will make it possible to use the *ph1b* mutant and wild type variants of the same (Mv9kr1) wheat genotype for interspecific hybridization programs to induce homoeologous recombination and later to stabilize the genome by elimination of the mutant 5B chromosome. The application of these genotypes will also avoid difficulties connected to multiple wheat genetic backgrounds during the agronomic evaluation of the introgression lines. The uniform wheat genetic background means further advantage when translocation chromosomes are used to map the introgressed alien chromosome segments for cloning genes with agronomical importance (Thind et al., 2017).

Morphological characterization showed that the plants containing the Norwich variant of *ph1b* mutant chromosome 5B in the Mv9kr1 background (Mv9kr1*ph1b*_N) had lower fertility than those of the Mv9kr1*ph1b*_K mutant, indicating that additional genetic modifications occurred in the Mv9kr1*ph1b*_N genotype. In line with this, flow cytometric chromosome analysis suggested that the chromosome 5B of the Norwich variant of Chinese Spring *ph1b* has lower DNA content as compared to the Kansas genotype. The size of the wild-type chromosome 5B in Chinese Spring was estimated as 870 Mbp (IWGSC International Wheat Genome Sequencing Consortium, 2014), and the population of this chromosome was located on a flow karyotype in a position typical for the chromosome 5B in hexaploid wheat with a wild-type karyotype (Doležel et al., 2021). Dunford et al. (1995) estimated the size of the 5B deletion in *ph1b* mutant as ~70 Mbp, and this region was further narrowed down to 59.3 Mbp with 1,187 genes by Martín et al. (2018). This ~6.8% reduction in the chromosome size resulted in the shift of the 5B population’s position toward a smaller DAPI fluorescence intensity (left of *x*-axis) on the flow karyotype. The fact that this shift was more pronounced in the Norwich variant *ph1b* mutant suggests that additional loss of 5B DNA content happened in this genotype. The smaller size of the Norwich variant of *ph1b* chromosome 5B was confirmed in the genotype Mv9kr1*ph1b*_N, which has a decreased fertility. These results are consistent with the previous observation that the inactivity of *Ph1* locus may result in karyotype instability in the *ph1b* mutant wheat (Sánchez-Morán et al., 2001).

Due to homoeologous synapsis and crossovers, the *ph1b* mutant wheat exhibited an increased number of homoeologous metaphase I associations, most frequently between A and D genome chromosomes, which resulted in the formation of intergenomic chromosome rearrangements (Sánchez-Morán et al., 2001). These intergenomic exchanges have most likely been accumulated generation by generation resulting in decreased fertility of the *ph1b* mutants relative to the wild-type genotypes as was observed earlier (Sears, 1977) and by the present study. As a future research direction, it would be helpful to develop new wheat *Ph1* mutant lines, with reduced homoeologous synapsis and crossover at meiosis, but which exhibit homoeologous crossovers in wheat-alien hybrids. The complex *Ph1* locus affecting both synapsis and crossover, possesses CDK2-like and a ZIP4 paralogue (*Tazip4-B2*) genes. It has been proposed, that *Ph1’s* function on synapsis is related to CDK2-dependent chromatine phosphoryllation (Martín et al., 2017), while ZIP4 is involved in the effect of *Ph1* on crossover formation (Martín et al., 2017; Rey et al., 2017). Recent improvements in CRISPR/Cas9 gene editing system allow the development of meiotically stable deletion mutants where the ZIP4 function is specifically knocked out to increase the crossover frequency without affecting the synapsis formation (Rey et al., 2018; Martín et al., 2021). Advances in wheat genetic transformation efficiencies makes it possible to achievable these goals (Hayta et al., 2021).

Flow karyotyping of the wild type and *ph1b* mutant wheat genotypes also indicated that a ~6.8% difference in the chromosome size allows discrimination of the deletion chromosome on a flow karyotype. This provides an opportunity for physical mapping of chromosomes based on the flow sorting of deletion chromosomes if deletion stocks for an entire chromosome are available (Svačina et al., 2019).

The increased frequency of wheat-alien chromosome associations and multivalent formation at meiotic metaphase I of wheat × alien F_1_ hybrids is a typical phenotype of the lines lacking *Ph1* locus (Qi et al., 2007; Moore, 2014; Naranjo and Benavente, 2015). In the present study, we used *Ae. biuncialis*, which is considered a rich source of genes for alien introgression breeding of wheat (Schneider et al., 2005; Farkas et al., 2014), to produce wheat-alien F_1_ hybrids to validate the promoting effect on homoeologous chromosome pairing of the new Mv9kr1*ph1b*_K genotype.

Logojan and Molnár-Láng (2000) reported a low frequency of meiotic pairing between wheat and *Ae. biuncialis* chromosomes in wild-type Mv9kr1—*Ae. biuncialis* F_1_ hybrids (ABDU^b^M^b^). The present work showed that the *ph1b* mutation in Mv9kr1 genetic background significantly increases the frequency of homoeologous metaphase I associations as compared to the wild-type genotype. An increased level of wheat-*Aegilops* chromosome pairing was also observed by Cifuentes et al. (2006) who investigated meiotic chromosome pairing in durum wheat × *Ae. geniculata* interspecific hybrids (2*n* = 4*x =* 28, ABU^g^M^g^) in the presence or absence of *Ph1* locus. Unfortunately, the genomic probes used by the authors did not allow discrimination between U and M genomes. In this study, we identified the M^b^ and U^b^ genome chromosomes by GISH and this allowed us to compare pairing affinity of constituent *Aegilops* genomes with the chromosomes of wheat. We found that the wheat chromosomes paired preferentially with the M^b^ genome chromosomes (3.55 w-M^b^ associations per cell) relative to U^b^ genome chromosomes (1.575 w-U^b^ associations per cell). Similar frequency of w-w and w-M^b^ homoeologous associations could be a consequence of high degree of homology between the M^b^-genome chromosomes and the corresponding chromosomes of wheat. The predominant pairing affinity of wheat chromosomes with the M^b^ genome chromosomes relative to U^b^ chromosomes are consistent with the earlier meiotic pairing analysis of F_1_ hybrids obtained by the crossing Chinese Spring *ph1b* mutant and Mv9kr1-*Ae. biuncialis* disomic additions 2M^b^, 3M^b^, 7M^b^, and 3U^b^ (Molnár and Molnár-Láng, 2010). Beside the fact that these monosomic wheat-*Aegilops* additions were heterozygous for the *ph1b* mutation and contained two copies of each wheat chromosomes, a tendency for increased level of wheat-*Aegilops* chromosome pairing were observed for 2M^b^ and 3M^b^ realtive to 3U^b^ chromosomes (Molnár and Molnár-Láng, 2010).

The chromosome pairing results are consistent with the previous investigations of the macro-level chromosome structure of wheat and M- and U-genomes of *Aegilops* by mapping conserved orthologous genes using single-gene FISH (Said et al., 2021) and COS markers (Molnár et al., 2013, 2016). These studies indicated close macrosyntenic relationships between the M-genome chromosomes with the corresponding chromosomes of wheat. On the other hand, the lower frequency of w-U^b^ metaphase I associations suggests larger structural differences between the U^b^ genome chromosomes and wheat. In fact, genetic mapping (Zhang et al., 1998; Edae et al., 2016, 2017), COS marker mapping on chromosome addition lines (Molnár et al., 2013) and single-gene FISH maps (Said et al., 2021) showed that U-genome of diploid *Ae. umbellulata* underwent multiple genome rearrangements during evolution resulting in synteny breaks in some chromosomes relative to wheat. While chromosomes 1 U, 2 U, 3 U, and 5 U remained more or less syntenic with wheat, chromosome 4 U contains regions homoeologous with wheat (w) chromosome groups 4, 5, and 6, 6 U homoeologous with w1, w2, w4, w6 and w7, while 7 U contains regions syntenic with w7 and w3 chromosomes. It is highly probable that these structural differences decreased the pairing affinity between U^b^ and wheat chromosomes in wheat- – *Ae. biuncialis* F_1_ hybrids.

Considering the differences in U- and M-genome chromosome structure, and their distinct affinity to pair with chromosomes of wheat, it may be concluded that *ph*-induced homeologous recombination is an effective strategy to transfer chromatin segments from all of the M^b^ chromosomes and from significant number of U^b^ chromosomes into wheat. In line with this, the feasibility of induced homoeologous chromosome pairing to transfer genes from U/M-genome *Aegilops* species into wheat has been demonstrated for *Ae. umbellulata* (Bansal et al., 2020) and *Ae. geniculata* (Kuraparthy et al., 2007).

The present study underlines the potential of the *ph*-based strategy when 29.16% of the Mv9kr1ph1b_K-*Ae. biuncialis* amphiploid plants contained intergenomic chromosome rearrangements. Wheat-*Aegilops* amphiploids thus obtained contain genetic variation from five *Ae. biuncialis* accessions originating from diverse geographical regions (Ivanizs et al., 2019) and presumably represent various allelic combinations for agronomically important genes. As these plants are homozygous for *ph1b* deletion, new wheat-*Aegilops* rearrangements can be produced in each new generation. These amphiploid genotypes may be used to generate new wheat-*Ae. biuncialis* chromosome translocations for wheat breeding through backcrossing with the wild type Mv9kr1 line. To conclude, the Mv9kr1*ph1b* mutant genotype developed in this work is an effective tool to facilitate alien gene introgression into hexaploid wheat.

## Data Availability Statement

The original contributions presented in the study are included in the article/Supplementary Material, further inquiries can be directed to the corresponding author.

## Author Contributions

IM: conceptualization, methodology, data curation, and project administration. IM, ET, LI, EG, AF, MS, PC, ÉS, KS-P, KK, and PK: investigation. IM and ÉS: resources. ET, IM, LI, and MS: visualization and writing—original draft preparation. IM, JD, and SG: writing—review. IM and JD: funding administration. All authors have read and approved the manuscript.

## Funding

This work has been supported by the Hungarian National Research, Development and Innovation Office (K135057, K119387, TKP2021-NKTA-06, and 2019–2.1.11-TÉT-2019-00074), by ERDF project Plants as a Tool for Sustainable Global Development (no.CZ.02.1.01/0.0/0.0/16_019/0000827), and the Marie Curie Fellowship Grant award AEGILWHEAT (H2020-MSCA-IF-2016-746253).

## Conflict of Interest

The authors declare that the research was conducted in the absence of any commercial or financial relationships that could be construed as a potential conflict of interest.

## Publisher’s Note

All claims expressed in this article are solely those of the authors and do not necessarily represent those of their affiliated organizations, or those of the publisher, the editors and the reviewers. Any product that may be evaluated in this article, or claim that may be made by its manufacturer, is not guaranteed or endorsed by the publisher.

## Footnotes

^1^ http://www.wgin.org.uk
