# Supplementary material for: Transfer of the ph1b Deletion Chromosome 5B From Chinese Spring Wheat Into a Winter Wheat Line and Induction of Chromosome Rearrangements in Wheat-Aegilops biuncialis Hybrids
Source: Front Plant Sci. 2022 Jun 13;13:875676. doi: 10.3389/fpls.2022.875676 (PMC9234525; doi:10.3389/fpls.2022.875676)

**Supplementary Material**

**Table S1**. The number of **F_1_** seeds obtained from crosses whith Mv9kr1 wheat.

|  | **No. of progenies derived from CS*ph1b*_K parent** | **No. of progenies derived from CS*ph1b*_N parent** |
| --- | --- | --- |
| **F_1_** | 108 | 128 |
| **BC_1_F_1_** | 43 | 106 |
| **BC_2_F_1_** | 70 | 80 |

**Table S2.** Segregation of the homozygous *ph1b* deletion in F_3_ and BC populations derived from the crosses of Kansas (K) or Norwich (N) variants of CS*ph1b* mutants with the winter wheat genotype Mv9kr1 as detected by molecular markers specific for the deletion region.

|  | **No. of progenies analyzed with *ph1b* specific markers** | | **No. of progenies with *ph1b* deletion** | |
| --- | --- | --- | --- | --- |
|  | **K** | **N** | **K** | **N** |
| **F_3_** | 84 | 90 | 19 | 34 |
| **BC_1_F_1_** | 35 | 60 | 7 | 7 |
| **BC_2_F_1_** | 50 | 50 | 6 | 8 |
| **BC_3_F_1_** | 87 | 85 | 16 | 21 |

**Table S3**. Purity of the chromosome 5B flow sorted from the wild type (Chinese Spring, Mv9kr1) and *ph1b* mutant (CS*ph1b*_K, CS*ph1b*_N, Mv9kr1*ph1b*_K, Mv9kr1*ph1b_*N) wheat genotypes.

| **Genotype** | **Purity (%) of sorted chromosome 5B** | **Contaminating chromosomes** | **No. of chromosomes investigated** |
| --- | --- | --- | --- |
| CS | 52.1% | 1B: 39.3%; 4A: 1.0%;  7B: 5.3%, 4B: 2.1% | 154 |
| Mv9kr1 | 57.9% | 1B: 39.4%; 4B: 1.9%, 6B: 0.8% | 157 |
| CS*ph1b*_K | 89.1% | 4B: 8.1%, 6B: 1.3%; 4A: 0.75%; 1B: 0.75% | 148 |
| CS*ph1b*_N | 97.2% | 4B: 2.0%; 4A: 0.8% | 147 |
| Mv9kr1*ph1b*_K | 85.8% | 4B: 11.6%; 4A: 2.6% | 120 |
| Mv9kr1*ph1b_*N | 98.5% | 4B: 0.75%; 4A: 0.75% | 140 |

**Table S4.** The PCR conditions and the primers’ sequences of molecular markers used for detection of *ph1b* deletion (Roberts et al. 1999).

| **Marker name** | **Forward primer** | **Reverse primer** | **T_a_ (˚C)** |
| --- | --- | --- | --- |
| Xpsr574 | ATCGCTCCTCTGCTTGCTTC | GACCGCCTGAAACCTCCC | 56 |
| Xpsr128 | AGCGTATATTCACGCGCTCC | CGTAAGAACTCCCCAGGGTTTG | 56 |
| XAWJL3 | TGGCACCCTCAATGTAGAC | GCTTGCCCATTTCACAAC | 56 |

**T_a_** means the annealing temperature of the primer pairs.

**Figure S1.** Representative pictures of 5B mitotic chromosome fractions flow-sorted from the wild type Chinese Spring **(A)** and Mv9kr1 **(B)** genotypes and from the CS*ph1b*_K **(C)**, CS*ph1b*_N **(D)**, Mv9kr1*ph1b*_K **(E)** and Mv9kr1*ph1b*_N **(F)** genotypes after FISH with DNA repeats pSc119.2 (green), Afa family (red) and 45S rDNA probe (yellow). The 5B chromosome located near to other B genome chromosomes on the DAPI vs. GAA-FITC dot plot flow karyotype of wild type Chinese Spring **(A)** and Mv9kr1 **(B)** genotypes, therefore the flow sorted fractions are strongly contaminated with the chromosomes 1B (#), 4B (*) and 7B (arrowhead). Because of the ph1b deletion, the chromosome 5B has lower DNA content and discriminated better from 1B, 4B and 7B **(C-F)** resulting in pure (85-98%) fractions after flow sorting.


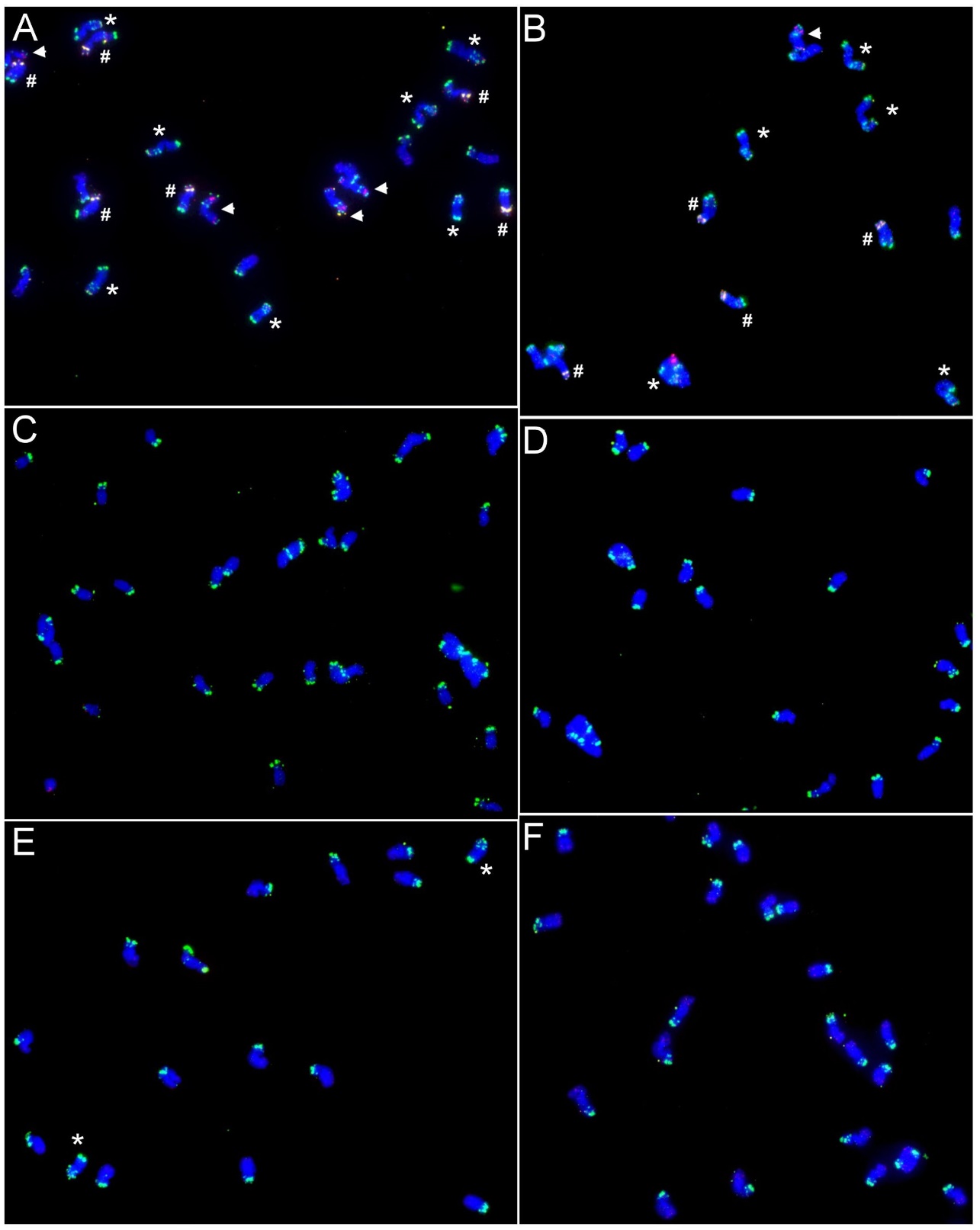

Supplement: Supplementary file 1 [file Data_Sheet_1.docx]
